# Carpal tunnel syndrome prediction with machine learning algorithms using anthropometric and strength-based measurement

**DOI:** 10.1371/journal.pone.0300044

**Published:** 2024-04-17

**Authors:** Mehmet Yetiş, Hikmet Kocaman, Mehmet Canlı, Hasan Yıldırım, Aysu Yetiş, İsmail Ceylan

**Affiliations:** 1 Department of Orthopedics and Traumatology, Faculty of Medicine, Kırşehir Ahi Evran University, Kırşehir, Turkey; 2 Department of Physiotherapy and Rehabilitation / Prosthetics-Orthotics Physiotherapy, Karamanoglu Mehmetbey University, Karaman, Turkey; 3 School of Physical Therapy and Rehabilitation, Kırşehir Ahi Evran University, Kırşehir, Turkey; 4 Department of Mathematics, Faculty of Kamil Özdağ Science, Karamanoglu Mehmetbey University, Karaman, Turkey; 5 Department of Neurology, Faculty of Medicine, Kırşehir Ahi Evran University, Kırşehir, Turkey; University of Illinois Urbana-Champaign, UNITED STATES

## Abstract

**Objectives:**

Carpal tunnel syndrome (CTS) stands as the most prevalent upper extremity entrapment neuropathy, with a multifaceted etiology encompassing various risk factors. This study aimed to investigate whether anthropometric measurements of the hand, grip strength, and pinch strength could serve as predictive indicators for CTS through machine learning techniques.

**Methods:**

Enrollment encompassed patients exhibiting CTS symptoms (n = 56) and asymptomatic healthy controls (n = 56), with confirmation via electrophysiological assessments. Anthropometric measurements of the hand were obtained using a digital caliper, grip strength was gauged via a digital handgrip dynamometer, and pinch strengths were assessed using a pinchmeter. A comprehensive analysis was conducted employing four most common and effective machine learning algorithms, integrating thorough parameter tuning and cross-validation procedures. Additionally, the outcomes of variable importance were presented.

**Results:**

Among the diverse algorithms, Random Forests (accuracy of 89.474%, F1-score of 0.905, and kappa value of 0.789) and XGBoost (accuracy of 86.842%, F1-score of 0.878, and kappa value of 0.736) emerged as the top-performing choices based on distinct classification metrics. In addition, using variable importance calculations specific to these models, the most important variables were found to be wrist circumference, hand width, hand grip strength, tip pinch, key pinch, and middle finger length.

**Conclusion:**

The findings of this study demonstrated that wrist circumference, hand width, hand grip strength, tip pinch, key pinch, and middle finger length can be utilized as reliable indicators of CTS. Also, the model developed herein, along with the identified crucial variables, could serve as an informative guide for healthcare professionals, enhancing precision and efficacy in CTS prediction.

## Introduction

Carpal Tunnel Syndrome (CTS) is the most common neuropathy of the upper extremities and is caused by compression of the median nerve under the flexor retinaculum of the wrist [1]. CTS is mainly due to fibrous hypertrophy of the synovial flexor sheath and repetitive wrist movements. The median nerve is damaged by mechanical compression and local ischemia in the carpal tunnel, and this causes changes in the myelin sheath and sometimes axon damage over time. Pain, pins and needles and loss of strength in the first 4 fingers, especially at night, are common symptoms [2]. Although entrapment neuropathies affect a small part of the nerve, they can cause significant physical, psychological and economic consequences [3]. The prevalence of CTS varies between 2.7% and 5.8% in the general adult population [4, 5]. Pain can radiate from the hands to the arms and shoulders. Personal risk factors associated with CTS include female gender, advanced age, pregnancy, obesity, thyroid diseases, diabetes, amyloidosis, trauma, and connective tissue diseases. Likewise, the working population is at risk for CTS. Work-related factors, especially repetitive movements, strenuous manual work, frequent wrist flexion, and hand-arm vibration have been implicated [6]. Although the most useful tests in the diagnosis of CTS are Tinel and Phalen tests, the most reliable objective method is electrodiagnostic tests. The appropriate medical practitioner must create a case history linked to the distinctive symptoms of CTS in order to diagnose patients with CTS. With the addition of findings such as thenar atrophy and sensory loss, the sensitivity of physical examination reaches 95.7% [7].

The most effective method for identifying peripheral nerve disorders is electrodiagnosis, which is also crucial in identifying CTS [8]. This method is useful for determining whether CTS exists and assessing its severity. Additionally, electrodiagnosis can be used to confirm differential diagnoses for neuropathies such as cervical radiculopathies [8, 9]. On the other hand, electrodiagnosis is invasive and could make the patient uncomfortable because it involves electrical stimulation and needle electromyography (EMG) during exams [10].

Machine-learning (ML)-based modeling is an emerging analysis tool that predictive model applications are its principal usage in medical research [11, 12]. Additionally, the classification of diseases, decision-making, and the creation of new treatment strategies can all be done using ML-based modeling [13, 14]. Although there has been explosive growth in machine learning-based medical research, CTS research is still relatively scarce. Some studies have explored predictive models for the diagnosis of CTS or classify CTS severity based on clinical data [15, 16]. Park et al. [15] performed seven ML models for classifying CTS severity. In a study by Faeghi et al. [16], sonographic images of the wrist were segmented, and using ML modeling, the accuracy of CTS diagnosis was evaluated. In another study, a deep learning algorithm was developed to improve the accurate identification of CTS [17]. Prior research suggests that some anthropometric factors may significantly contribute to the compression of the median nerve within the carpal canal. A recent meta-analysis indicates that a square-shaped wrist is a predictor for CTS in both males and females [18]. Mondelli et al. [19] assert that the wrist-palm ratio could aid in supporting the diagnostic hypothesis of severe CTS. Additionally, another study [20] suggests that greater hand volume, wrist circumference, and a lower palm length to proximal palm width ratio may be anthropometric risk factors in CTS. Morever, it has been noted that CTS can lead to a reduction in hand grip strength, which can be attributed to various underlying factors [21, 22]. To the best of our knowledge, there is no study that has investigated the diagnostic accuracy of CTS with ML-based methods based on anthropometric and strength measurements. A comprehensive investigation of anthropometric and strength measurements using ML algorithms may improve the understanding and management of CTS. Therefore, this study aimed to evaluate anthropometric and strength measurements to determine CTS using ML algorithms. We also identified the importance of variables in the performance of the ML-based diagnosed CTS.

## Materials and methods

### Study design

The study, designed as a cross-sectional case-controlled study, was conducted with 56 individuals diagnosed with CTS and 56 healthy, asymptomatic individuals. The ethics committee approval required for the research was obtained by the Ethics Committee of Kırşehir Ahi Evran University (Decision number: 2022-17/158). This study was carried out between November 1, 2022, and April 13, 2023. The present study was conducted in accordance with the Declaration of Helsinki. Written and verbal informed consent forms were obtained from all participants before starting the study.

### Participants

The study included 56 patients who applied to Kırşehir Ahi Evran University Orthopedics and Traumatology Polyclinic with CTS complaints and were diagnosed with CTS by specialist physicians, and 56 healthy individuals who were examined by specialist physicians. Inclusion criteria for the group diagnosed with bilateral CTS were: pain radiating to the palmar face of the hand or numbness; at least one of the phalen or carpal compression tests was positive in physical examination; symptoms had persisted for at least 3 months; and CTS was diagnosed electrophysiologically. Individuals without hand, wrist, or upper extremity pathology were included in the healthy group. Those who have been operated on for CTS, those who have undergone steroid injection, medical treatment, and physical therapy for CTS, thyroid dysfunctions, diabetes mellitus, pregnancy, and connective tissue diseases considered secondary causes of CTS; Also, those with other diseases that may affect the symptoms of the disease and cause neck and arm pain (cervical disc herniation, rotator cuff syndrome, epicondylitis, de Quervain’s tenosynovitis, Dupuytren’s contracture, history of wrist fracture, arthritis, more proximal upper extremity entrapment neuropathies, polyneuropathy, peripheral nerve injury, fibromyalgia syndrome) were not included in the study.

### Measurements

Before starting the study, age, height, weight, Body Mass Index (kg/m^2^), gender, sides with CTS, and dominant extremities of the participants were recorded. All evaluations were made by considering the dominant sides of individuals with bilateral CTS and healthy individuals.

Electromyographic examinations were performed with EMG device (MEB-9200K, Nihon Kohden Corporation, Tokyo, Japan). In sensory nerve conduction study, With the stimulation of the sensory branch of the median nerve from the 2nd finger and the sensory branch of the ulnar nerve from the 5th finger, sensory distal latency, action potential amplitude of the sensory nerve, and sensory nerve conduction velocity in the finger-wrist segment were examined. In motor transmission operation, Median and ulnar nerve distal motor latency (DML), compound muscle action potential (CMAP) amplitude, and wrist-elbow motor nerve conduction velocity parameters were investigated. The electrophysiological examination of all patients was performed by a single physician at room temperature. Normal reference values in our laboratory are: median motor distal latency ≤ 4ms, CMAP ≥4, and motor conduction velocity ≥50 m/s, sensory peak latency ≤3.2, sensory amplitude ≥12, sensory conduction velocity ≥50 m/s. According to the results of the electrophysiological examination, the patients were divided into three groups as mild, moderate and advanced CTS. Decrease in sensory conduction velocity only mild group, DML increase in median nerve with deceleration in sensory conduction velocity middle group; Inability to receive sensory conduction, increased DML in the median nerve, or no motor conduction recording was classified as the advanced group.

Hand Breadth was evaluated according to the standards defined by Singh and Bhasin (maximal distance between the heads of the second and fifth metacarpal bone) [23]. Wrist circumference was measured by wrapping the tape measure around the wrist, passing through the styloid processes of the radius and ulna. For the measurement of the length of the middle finger, the middle point on the proximal line (plica digitopalmaris) that separates the root of the finger from the palm of the hand was taken as the basis and was also evaluated using the distance between the tip of the middle finger [24].

The Hand Shape Index is the ratio of palm width to hand length and is calculated with the formula: 100 × palm width (mm) / hand length (mm) [25]. The Thenar Index is a percentage that indicates the ratio of thenar area to hypothenar area and is calculated using the formula: thenar area (mm^2^) / hypothenar area (mm^2^) X 100. The digit index is the ratio of the length of the third finger to the length of the hand and is calculated with the formula: 100 × third finger length (mm) / hand length (mm) [25]. The wrist index is the ratio of the wrist’s medio-lateral diameter to the dorso-volar diameter and is calculated by the formula: medio-lateral diameter (mm) / dorso-volar diameter (mm) [25].

The grip strength of dominant hand was measured using a standard adjustable digital handgrip dynamometer (Baseline Digital Smedley Hand Dynamometer). The handgrip strength was measured as follows: (a) Each subject was tested while sitting comfortably on a chair without arm rest, with his or her back leaned against the chair; (b) Each subject was instructed to sit with their hips and knees flexed at 90°, shoulders adducted and neutrally rotated, elbow flexed at 90°, forearm rotation at 0°, wrist between 0° and 30° of dorsiflexion and between 0° and 15° of ulnar deviation. Pinch strength was measured with pinchmeter (Jamar Digital Pinchmeter 50 LB) by tip (two-point) pinch, key (lateral) pinch, and palmar (three-jaw chuck) pinch. Tip pinch is thumb tip to index fingertip. Key pinch is thumb pad to lateral aspect of middle phalanx of index finger. Palmar pinch is thumb pad to pads of index and middle fingers. For each strength test the scores of three successive trials were recorded for dominant hand.

### Data analysis

In statistical analysis process, the results are represented as *mean* ± *standard deviation* for continuous variables and frequencies with percentages for categorical variables. In order to carry out the signifance tests between healthy and patient groups, the independent samples t-test is applied with *α* = 0.05 level. R and IBM SPSS (version 24.0) softwares have been used to conduct statistical analyses on the participants’ demographic variables.

### Model training and validation

In the process of developing machine learning models, the data has been preprocessed before the model fit procedure. This procedure resulted in the identification of missing, incorrect, and inconsistent data. To find outliers, box plots and z-scores were used. Correlation values have been used to explore the potential of multicollinearity. To obtain a more homogeneous set of data, centering and scaling have been utilized to eliminate unit disparities in the data. In this way, continuous variables have become zero mean and unit standard variance. Following the preprocessing steps, the dataset has been randomly split into a training and test set at a percentages of 65:35. A threefold cross validation with five repeats method has been used to provide a more generalizable result.

Several notable R packages including caret, tidymodels and tidyverse have been employed to implement machine learning algorithms. Support vector machines (with linear, polynomial and radial kernel), random forests (RF), neural networks (multi layer perceptron), eXtreme gradient boosting (XGBoost) have been employed in the study. Parameter adjustments have been made through random and/or grid search as when the models have being fitted to the training data. The test data has been used to assess the ML algorithms that have been developed using the optimal parameters determined in the cross validation data. As evaluation criteria for the model, accuracy, F1-measure, and kappa coefficient have been used. These criteria are defined based on the confusion matrix presented as follows (Table 1).

**Table 1:** The general representation of a classic confusion matrix.

		Predicted	
		Positive	Negative
**Actual (Truth)**	**Positive**	True Positive (TP)	False Negative (FN)
	**Negative**	False Positive (FP)	True Negative (TN)

The accuracy, Kappa and F-score, which are the most widely used measures in the literature, can be defined based on confusion matrix as follows:

**Accuracy**: Accuracy is defined as the ratio of correctly identified cases (including true positives and true negatives) compared to all cases and calculated based on the following formula.


$$\text{Accuracy}=\frac{\left(TP+TN\right)}{\left(TP+TN+FP+FN\right)}$$

The accuracy ratio ranges between [0,1] or percentage equivalent [0,100] and increases in parallel with the model prediction ability.

**F-score (or F1-score)**: The F1-score is a more robust alternative to the accuracy criterion and is particularly suitable for binary classification in cases of class imbalance or when false positives and false negatives are weighted in different amountsIt corresponds essentially to the harmonic mean of the precision and recall values. Likewise to the accuracy criterion, it has a range of [0,1] and increases proportional to the model performance and defined by using the confusion matrix components as follows:

$$\text{F-score}=2\times \frac{\text{Precision}\times \text{Recall}}{\text{Precision}+\text{Recall}}$$

where

$$\text{Precision}=\frac{TP}{\left(TP+FP\right)}$$

$$\text{Recall}=\frac{TP}{\left(TP+FN\right)}$$

**Kappa (κ) statistic:** The Kappa statistic (also known as Cohen’s Kappa) was originally proposed to evaluate the agreement between two raters, but is more commonly used in the machine learning literature as a measure of accuracy by chance. It is essentially employed to determine the extent to which model predictions perform better than random predictions. Similar to the F1-score, it is a more robust measure than the accuracy criterion and takes values in the range [-1,1]. A Kappa value of -1 is interpreted as a complete disagreement between model prediction and true values, a value of 1 as a complete agreement between model prediction and true values, while a value of 0 is interpreted as a performance equivalent to random prediction. Kappa criterion (κ) is calculated by using the observed agreement (*p*_*o*_), expected agreement (*p*_*e*_) and the number of cases (N) and defined as follows:

$$\kappa =\frac{\left({\text{p}}_{\text{o}}-{\text{p}}_{\text{e}}\right)}{\left(1-{\text{p}}_{\text{e}}\right)}$$

where

$$p_{o}=\frac{TP+TN}{TP+TN+FP+FN}$$

$${\text{p}}_{\text{e}}=\frac{\left(\text{TP}+\text{FN}\right)\left(\text{TP}+\text{FP}\right)+\left(\text{FP}+\text{TN}\right)\left(\text{FN}+\text{TN}\right)}{{\text{N}}^{2}}$$

**Fig 1:**
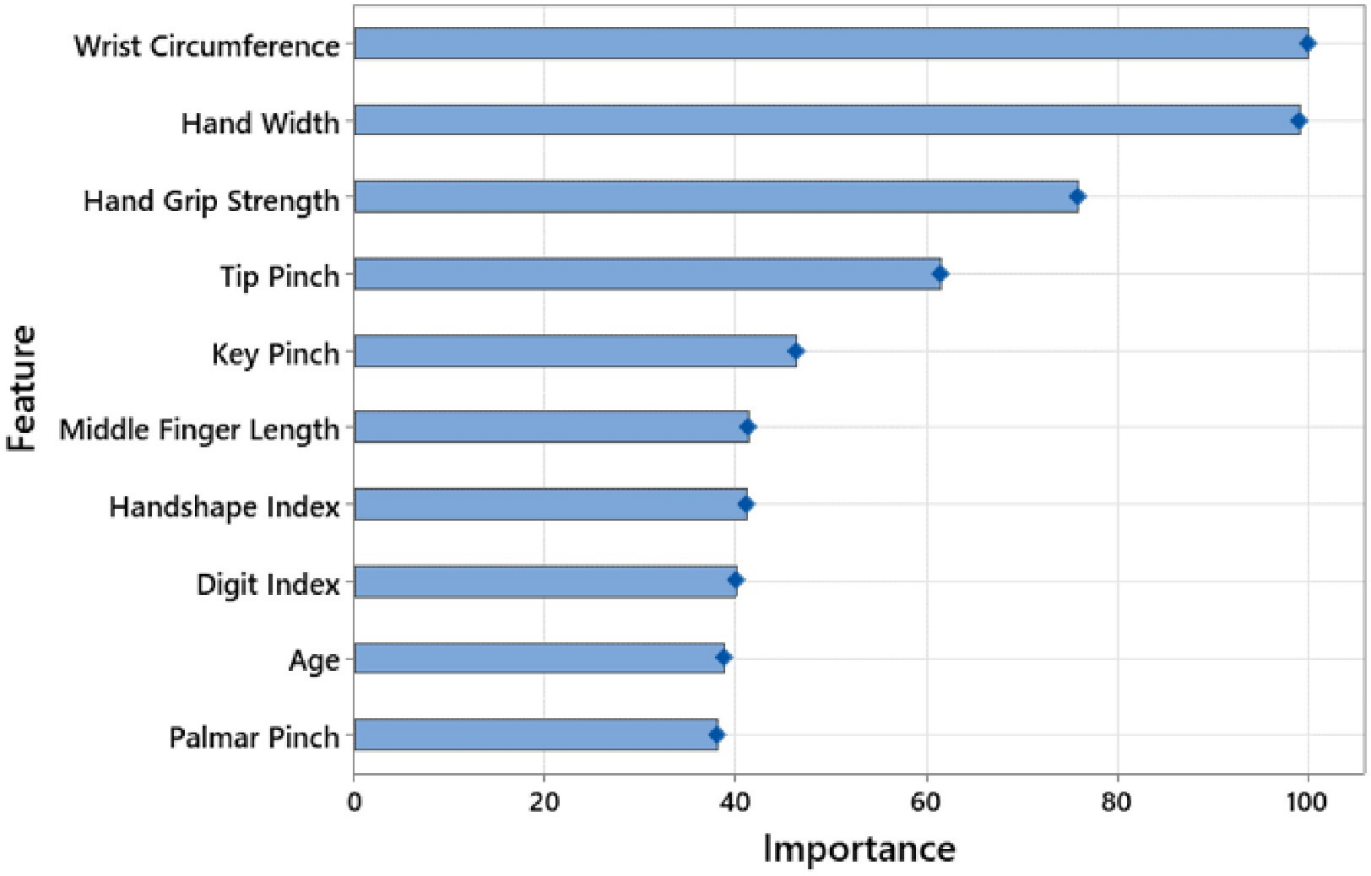
Top 10 features based on hand measurements (Random forests).

**Fig 2:**
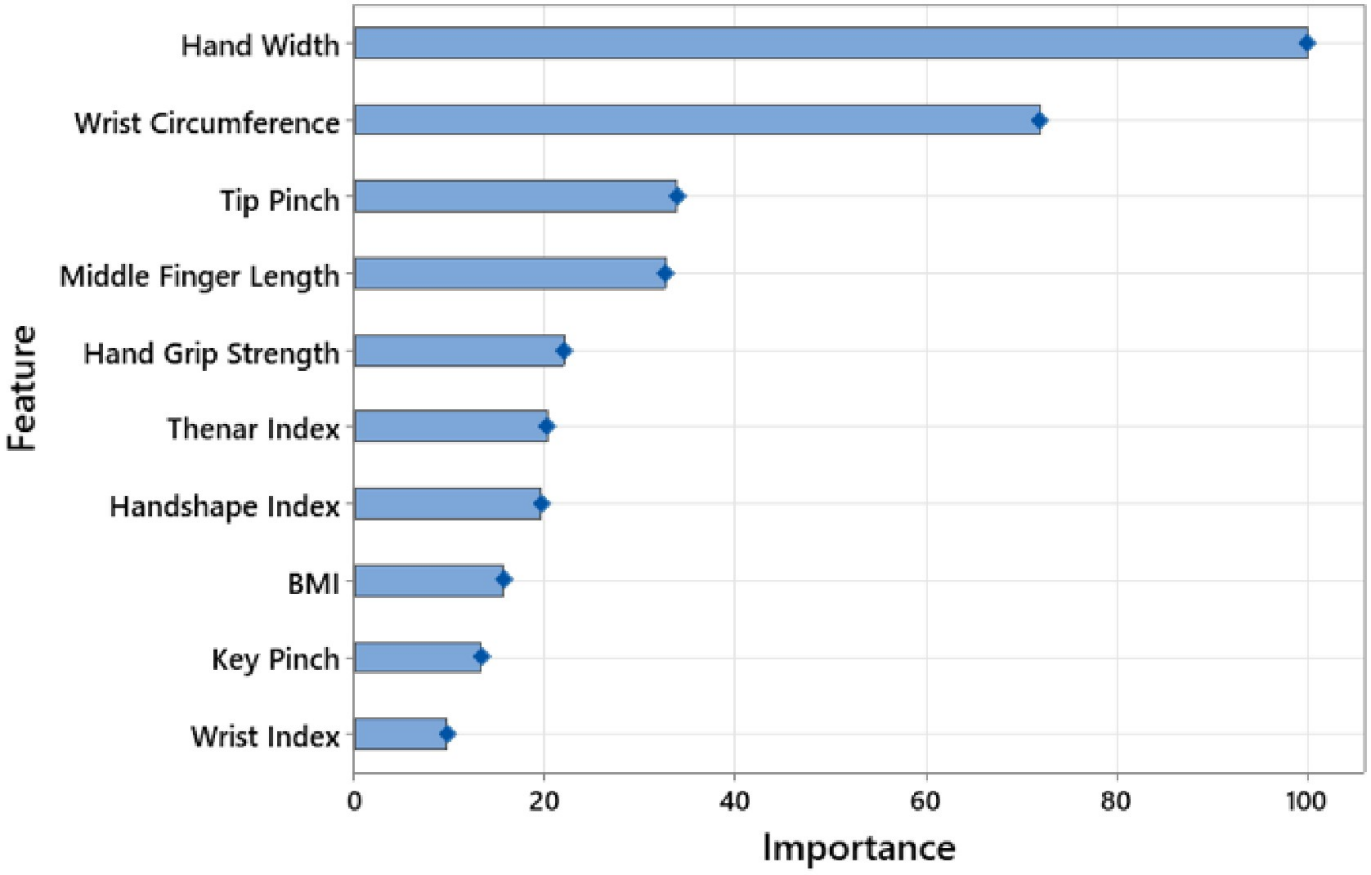
Top 10 features based on hand measurements (XGBoost).

As a results of consideration the aforementioned criteria, RF and XGBoost have been determined to be the top two models based on the performance values listed in Table 2. Table 3 provides the confusion matrices upon these two models. Variable importance analysis has been carried out to establish the relative importance of the significant factors for disease detection. Results for the RF and XGBoost models, respectively, are shown in Figs 1 and 2, along with their ranking of importance.

**Table 2:** Statistical significance results based on dominant hand measurements.

	**Group**				
	**Healthy** **(n = 56)**		**Patient** **(n = 56)**		
	**Mean** **[95.0% CI]**	**SD**	**Mean** **[95.0% CI]**	**SD**	**p**
**Age**	48.2 [43.4, 52.6]	4.0	49.1 [42.6, 54.3]	5.5	0.137
**BMI**	28.8 [27.6, 30.0]	4.4	30.3 [28.9, 31.6]	5.0	0.101
**Digit Index**	40.2 [38.4, 41.9]	6.4	43.1 [42.7, 43.4]	1.3	**0.001**
**Hand Grip Strength**	30.8 [27.7, 33.8]	6.4	23.3 [21.8, 24.8]	5.6	**<0.001**
**Hand Shape Index**	46.9 [45.4, 48.5]	5.8	45.3 [44.2, 46.3]	3.9	0.083
**Hand Width**	58.3 [56.4, 60.1]	6.8	64.9 [62.8, 67.1]	8.1	**<0.001**
**Key Pinch**	9.3 [8.4, 10.1]	3.2	7.4 [6.5, 8.4]	3.6	**0.004**
**Middle Finger Length**	76.1 [75.0, 77.1]	4.0	71.2 [67.4, 75.0]	4.3	**0.015**
**Palmar Pinch**	8.1 [7.4, 8.8]	2.7	6.7 [5.9, 7.5]	3.1	**0.014**
**Thenar Index**	1.7 [1.6, 1.7]	0.3	1.7 [1.7, 1.8]	0.3	0.195
**Tip Pinch**	13.4 [12.1, 14.7]	4.9	9.0 [7.8, 10.2]	4.6	**<0.001**
**Wrist Circumference**	189.1 [180.0, 198.2]	13.9	178.7 [171.9, 185.5]	15.3	**0.048**
**Wrist Index**	1.4 [1.3, 1.4]	0.1	1.4 [1.3, 1.4]	0.1	0.442
	**Count**	**Percentage**	**Count**	**Percentage**	**p**
**Gender (Female)**	33	47.8	36	52.2	0.564
**Gender (Male)**	23	53.5	20	46.5	

SD: Standart Deviation, BMI: Body Mass Index, p<0.05

**Table 3:** The results of machine learning algorithms based on the dominant hand measurements.

	Training	Testing		
	Accuracy (%)	Accuracy (%)	F1	Kappa
**Support Vector Machines (Polynomial)**	94.7	76.316	0.756	0.526
**Support Vector Machines (Radial)**	94.3	73.684	0.722	0.473
**Random Forests**	95.8	**89.474**	**0.905**	**0.789**
**Neural Networks (MLP)**	95.6	78.947	0.789	0.578
**eXtreme Gradient Boosting (Linear)**	96.2	**86.842**	**0.878**	**0.736**

## Results

Table 2 summarizes the main statistical findings according to the participant characteristics. This table indicates whether the clinical and demographic features varied between the healthy and patient groups. It can be inferred that there is a balanced distribution among the male and female individuals when examining the frequencies on a group level. Digit Index, Hand Grip Strength, Hand Width, Key Pinch, Middle Finger Length, Palmar Pinch, Tip Pinch and Wrist Circumference variables highlight statistically significant differences between patient and healthy individuals when continuous variables are analyzed (p<0.05). The remaining variables’ difference is not statistically significant (p>0.05).

### Binary classification

Considering that the aim of the study is to predict patients with CTS and healthy individuals with the least error, high accuracy, F1-measure and kappa value will reveal the best model. High test performance along with high training performance are required while identifying the best model. According to these criteria, RF and XGBoost algorithms, which have the accuracy values of 95.8% and 96.2%, respectively, have been found to be the best in training performance. On the other hand, the two top models in test performance have been RF (89.474% accuracy, 0.905 F1-score, and 0.789 kappa value) and XGBoost (86.842% accuracy, 0.878 F1-score, and 0.736 kappa value) (Table 3). The confusion matrices presented in Table 4 further confirm a strong agreement between the actual response values and the predicted values, supporting the result that both models demonstrate convincing performance. In order to guarantee the possible reproducibility of the study results, the parameter space and the optimal values considered for each model are presented in Table 5.

**Table 4:** The confusion matrices corresponds to the random forests and XGBoost algorithms.

Algorithms	Predicted	Actual	
		Yes	No
**Random Forests (RF)**	Yes	**19**	**4**
	No	**0**	**15**
**eXtreme Gradient Boosting (XGBoost Linear)**	Yes	**18**	**4**
	No	**1**	**15**

**Table 5:** The ranges of parameters and optimal values corresponding to the each model.

Model	Range of Parameters	Optimal Values
**Support Vector Machines (Polynomial)**	Cost: [2^−5^, 2^10^] Polynomial Degree: [1, 3] Scale: [10^−5^, 10^2^]	9.288787 3 1.560586
**Support Vector Machines (Radial)**	Cost: [2^−5^, 2^10^] Sigma: [10^−10^, 1]	836.6652 0.4629227
**Random Forests**	The Number of Randomly Selected Predictors [1, 13]	4
**Neural Networks (MLP)**	Hidden units: [0, 100] Weight decay: [10^−5^, 10]	20 0.00206593
**eXtreme Gradient Boosting (Linear)**	L2 Regularization: [10^−5^, 1] L1 Regularization: [10^−5^, 1] The Number of Boosting Iterations: [1, 100] Learning Rate: [*up to* 3]	0.03454034 0.0006712936 55 1.096612

Since these two models have variable intrinsic importance index, variable importance evaluation has been carried out. Figs 1 and 2 present the findings of the analysis. Upon inspection of Figs 1 and 2, wrist circumference, hand width, hand grip strength, tip pinch and key pinch are the five most important features in the RF model, while the wrist circumference, hand width, hand grip strength, tip pinch and middle finger length have been the five most essential features in the XGBoost model. It is seen that the importance ratings of the machine learning algorithms and the statistical significance values substantially conform to each other. In this regard, it is apparent that the proposed model provides convincing results in terms of both traditional statistical analysis and artificial intelligence performance.

## Discussion

In this study, as with electrodiagnostic techniques, we used ML-based modeling approaches to describe CTS based on anthropometric features. All ML models provided over 70% accuracy. On the other hand, the RF and XGBoost models performed best. Furthermore, using variable importance calculations specific to these models, it was determined that the most important variables for predicting CTS were wrist circumference, hand width, hand grip strength, tip pinch, key pinch, and middle finger length.

CTS has a wide range of signs and symptoms. The diagnosis of CTS is made by the patient’s history and physical examination. Electrodiagnostic evaluation is used to confirm the diagnosis and determine its severity. Although electrodiagnostic evaluations provide 85% sensitivity and 95% specificity for diagnosing CTS, they are invasive and very uncomfortable for many patients [26, 27]. ML models have been used in previous studies to assess CTS. In a study by Faeghi et al. [16], sonographic images of the wrist were collected from the CTS and control groups. The images were segmented, and using ML modeling, the accuracy of CTS diagnosis was evaluated. They claimed that when the computer-aided diagnosis was used, radiologists’ diagnostic precision increased [16]. In another study, for CTS detection, Sayin et al. [28] used four ML algorithms (support vector machine, naive Bayes, classification tree, and artificial neural network). They reported that naive bayes yielded better performance than all the other methods in the diagnosis of CTS, followed by support vector machine with a 91.0% CTS detection score. Park et al. [15] performed seven ML models for classifying CTS severity. According to the study, the RF and XGBoost models showed better performance, and the numeric rating scale of pain was the most important variable, and body mass index was the second most important. Similar to the findings of Park et al. in the present study, RF and XGBoost models showed the best performance in the identification of CTS.

Previous findings indicate that some anthropometric factors play an important role in the compression of the median nerve in the carpal canal [18, 25]. A study assessed the association of carpal tunnel syndrome with anthropometric characteristics of the hand and body mass index (BMI) as independent risk factors. It was found that wrist index and wrist width were significantly higher in CTS patients than in the control group. Also, the study revealed that wrist index, BMI, and the ratio of hand length to body height were independent risk factors for CTS [25]. Moghtaderi et al. [29] identified being female, gender, obesity, and possessing a square wrist as independent risk factors for CTS. Moreover, the study noted a reduction in wrist circumference with escalating disease severity in individuals with CTS in comparison to their healthy counterparts. Another study identified independent risk factors for CTS as wrist index, BMI, and the ratio of hand length to body height. Also, this study concluded that individuals with CTS displayed shorter finger length and wider hands compared to their healthy counterparts [25]. In parallel with previous studies, in the present study, wrist circumference, hand width, and middle finger length anthropometric measurements were determined as the most important parameters in the identification of CTS according to RF and XGB machine learning models, which were determined to show the best performance. The anthropometric features of the hand can play a role in influencing hand biomechanics. Studies indicate that a more square-shaped hand requires increased volar extension or flexion for a specific movement, potentially contributing to median nerve compression [30, 31]. Additionally, shorter hands may experience heightened tension on finger flexor tendons during pinching or clenching, leading to increased pressure within the carpal tunnel [31]. In the current study, the most important parameters predicting CTS can be elucidated through the aforementioned mechanisms. Nevertheless, more precise evidence is warranted to draw clearer conclusions. In addition, the finding that some anthropometric measurements such as wrist circumference, hand width, and middle finger length were found to be predictive in the identification of CTS in the current study may emphasize the importance of paying attention to the anthropometric characteristics of the hand in CTS.

CTS can trigger a reduction in hand strength due to a multitude of underlying factors. Grip and pinch strength deterioration linked to muscle tension in CTS patients may stem from the impairment of metacarpophalangeal (MCP) joint flexion potency attributed to the de-innervation of the first and second lumbricals. This is particularly significant as the extrinsic flexors receive higher innervation in the forearm [32]. The intrinsic muscles, encompassing palmar interossei, dorsal interossei, and lumbrical muscles, counterbalance proximal interphalangeal (PIP) and distal interphalangeal (DIP) joint flexion while facilitating precision grip and pinch by flexing the MCP joints while concurrently extending the IP joints. A common observation among CTS patients is the heightened tautness of lumbrical muscles, which subsequently engenders escalated resistance or drag during extension. This, in turn, thwarts robust flexion of the DIP and PIP joints. Moreover, another contributor to the decline in strength might be the frequently encountered sensory disruption in CTS. This sensory impairment has the potential to curtail the precision of force regulation in affected individuals [32, 33]. In the current study, in the analyses performed using model-specific variable importance calculations, hand grip strength, tip pinch, and key pinch were found to be important variables in the prediction of CTS according to the two best-performing models, RF and XGBoost. This result supports the information reported in the literature that there is a change in grip and pinch strength in CTS.

The present study possesses certain limitations that should be acknowledged. Firstly, our analysis focused exclusively on the dominant side of individuals diagnosed with bilateral CTS. Notably, CTS severity is commonly classified into three levels. However, our study did not differentiate individuals based on the severity of CTS. Therefore, we suggest that future investigations should consider conducting separate analyses for individuals, taking into account the severity of CTS, and extending the examination to include their non-dominant sides.

## Conclusion

This study presents the performance of top machine learning models (including support vector machines, neural networks, RF, and XGBoost) developed to identify important factors affecting carpal tunnel syndrome and to accurately predict individuals with this disease.

In the experiments performed, as a result of various criteria (accuracy, F1-score, and kappa), RF and XGBoost algorithms were found to be superior in both training and test performance. In addition, the most important variables were found to be wrist circumference, hand width, hand grip strength, tip pinch, key pinch, and middle finger length by using the variable importance calculations intrinsic in these models. Furthermore, this result was found to be compatible with statistical analyses. Through this study, it is considered that the use of machine learning methods as well as the classical statistical approaches will improve the decision-making processes of the experts in the field, as well as better understanding of different diseases by modeling them in a similar way.
